# Ultrasound-guided retro-superior costotransverse ligament space block (RSSB) versus subcostal transversus abdominis plane block (TAPB) for postoperative analgesia in gastric cancer patients undergoing laparoscopic gastrectomy : a prospective randomized controlled trial

**DOI:** 10.1186/s12871-026-03700-7

**Published:** 2026-02-23

**Authors:** Yuge Liu, Wei Zhao, Xueqiu Zhang, Beibei Yu, Xi Chen, Yibo Huang, Ming Yan

**Affiliations:** 1 Department of Anesthesiology, Affiliated Hospital of Xuzhou Medical University, Xuzhou, Jiangsu 221000 China; 2 Department of Thoracic Surgery, Affiliated Hospital of Xuzhou Medical University, Xuzhou, 221000 Jiangsu China; 3 School of Anesthesiology, Xuzhou Medical University, Xuzhou, 221000 Jiangsu China

**Keywords:** Nerve block, Anesthesia, Gastrectomy, Postoperative pain, Ultrasonography

## Abstract

**Background:**

Retro-superior costotransverse ligament space block (RSSB) is a new variant of thoracic paravertebral block (TPVB), while subcostal transversus abdominis plane block (TAPB) is a commonly used analgesic technique in laparoscopic surgery. This study aimed to compare the effects of RSSB and TAPB on postoperative analgesia and quality of postoperative recovery in patients with gastric cancer undergoing laparoscopic gastrectomy. We hypothesized that RSSB provides more comprehensive analgesia and promotes postoperative recovery than TAPB.

**Methods:**

This prospective randomized controlled trial enrolled 60 patients with gastric cancer undergoing laparoscopic gastrectomy. Eligibility criteria included age 18–85 years, body mass index (BMI) 18.5–28 kg/m², American Society of Anesthesiologists (ASA) physical status I–III, and scheduled elective laparoscopic gastrectomy. Patients were assigned to two groups in a 1:1 random manner. The RSSB group received bilateral injections of 40 mL 0.375% ropivacaine at the T7–8 level; the TAPB group received the same volume and concentration of ropivacaine (40 mL total, 20 mL per side) along the subcostal anterior axillary line. The primary outcome was the area under the curve (AUC) of numeric rating scale (NRS, 0 to 10) during 24 h postoperatively.

**Results:**

A total of 52 patients were included in the analysis. NRS-AUC within 24 h postoperatively was significantly lower in the RSSB group compared to the TAPB group (mean (SD) AUC: 48.3 (24.0) vs. 75.4 (35.7), *P* = 0.002). The neural block spread range in the RSSB group was significantly wider than in the TAPB group (mean (SD) superficial spread segments: 5.2 (2.1) vs. 3.0 (0.7), *P* < 0.001). Meanwhile, the time to first Patient-controlled analgesia (PCA) bolus request was markedly prolonged in the RSSB group compared with the TAPB group (mean (SD) 480.6 ± 385.8 vs. 249.1 ± 256.4, *P* = 0.034). Additionally, within the first 24 h postoperatively, the RSSB group scored higher on the Quality of Recovery-15 (QoR-15) scale than the TAPB group (mean (SD): 122.4 (7.1) vs. 115.0 (16.4), *P* = 0.039). No statistically significant differences were observed between the two groups in other secondary outcome measures.

**Conclusions:**

This study demonstrates that RSSB provides better postoperative analgesia than subcostal TAPB and improves early postoperative recovery quality in patients undergoing laparoscopic gastrectomy.

**Trial registration:**

ChiCTR2500096679. Registered 1 February 2025 (prospectively registered).

**Supplementary Information:**

The online version contains supplementary material available at 10.1186/s12871-026-03700-7.

## Introduction

Gastric cancer remains a major global health burden, characterized by high morbidity and mortality despite advances in surgical techniques [1, 2]. Although laparoscopic gastrectomy is minimally invasive, many patients still experience significant postoperative pain, which hinders early mobilization and delays recovery under Enhanced Recovery After Surgery (ERAS) protocols [3–5]. Opioid analgesia is commonly used but is associated with adverse effects such as nausea, ileus, and respiratory depression, prompting the adoption of regional anesthesia within multimodal analgesic strategies [6].

The subcostal TAPB is frequently applied to reduce abdominal wall pain and opioid consumption during upper abdominal surgery [7]. However, its effect is limited to somatic innervation and insufficient for visceral pain control [8]. To address this limitation, the RSSB, a novel modification of thoracic paravertebral block, targets the posterior costotransverse compartment, which communicates with spinal nerve branches and the sympathetic chain, potentially providing both somatic and visceral analgesia [9, 10].

Currently, there are only limited case reports on the application of RSSB in laparoscopic gastrectomy [9], and no randomized controlled trials have directly compared RSSB with subcostal TAPB. Instead, most existing studies have focused on comparisons between TAPB and intertransverse process block (ITPB) or erector spinae plane block (ESPB) [11, 12]. However, as a novel variant of TPVB, the analgesic advantages of RSSB have not been fully verified. Therefore, this study provides new clinical evidence for perioperative analgesia in laparoscopic gastrectomy by directly comparing the analgesic effects and impacts on postoperative recovery of these two techniques. We hypothesized that RSSB would provide more comprehensive pain relief and enhance early recovery.

## Methods

### Ethics and study design

This single-center, prospective randomized controlled trial followed the principles of the *Declaration of Helsinki* strictly and was reported according to CONSORT guidelines. The study protocol received ethical clearance from the Ethics Committee of the Affiliated Hospital of Xuzhou Medical University (XYFY2024-KL649-01), and written informed consent was obtained from each participant. In compliance with clinical research standards, the trial was also registered in the Chinese Clinical Trial Registry (ChiCTR2500096679) on February 1, 2025 with the first patient enrolled on March 1, 2025. Link to the trial’s registration documents: https://www.chictr.org.cn/index.html

### Participants 

Included patients were aged 18–85 years with gastric cancer, were classified as ASA physical status II–III, with BMI 18.5–28 kg/m² (including critical values), and scheduled for elective laparoscopic gastrectomy. Exclusion criteria included: contraindications to regional anesthesia (coagulation disorders; bacteremia, sepsis, or puncture site infection; allergy to ropivacaine or lidocaine); pre-existing chronic pain, long-term opioid use; liver cirrhosis or renal impairment (estimated glomerular filtration rate < 30 mL/min/1.73 m²); unplanned conversion to open surgery or postoperative ICU admission for mechanical ventilation; patient non-cooperation (inability to evaluate outcome measures); pregnancy, lactation, or participation in other clinical trials. Withdrawal criteria included: surgery cancelation or voluntary withdrawal by the patient; severe hemodynamic disorders or life-threatening complications; prolonged hospital stay due to severe postoperative complications (ICU admission); death within 24 h after surgery.

### Randomization and blinding

Due to inherent differences in the operational positions and sites of the two blocking techniques, this trial could not implement blinding for patients and the anesthesiologists performing the blocks. However, we adopted a strict evaluator blind method. One researcher (X-C), unaware of group assignments, obtained informed consent and collected baseline data in the ward one day before surgery. After enrollment, another researcher (B-Y) used SPSS 25.0 software (IBM, Chicago, USA) to randomly assign patients to the RSSB group or TAPB group at a 1:1 ratio via a computer-generated simple randomization table. Group assignments were sealed in sequentially numbered opaque envelopes for blinding. Before surgery, the researcher (B-Y) opened the envelope to prepare study drugs. All nerve blocks were performed by an experienced anesthesiologist (W-Z). During the intervention, the responsible anesthesiologist left the operating room and returned after the block was completed. Follow-up data were collected and analyzed by other researchers (Y-L, X-Z, Y-H). Intraoperative anesthesiologists, follow-up evaluators, data analysts were blinded.

### General anesthesia management and postoperative analgesia

Anesthesia followed standardized institutional protocols. Upon entering the operating theater, each patient was equipped with standard monitoring devices, including a five-lead electrocardiogram, noninvasive blood pressure cuff, and pulse oximeter, alongside radial artery cannulation for invasive arterial pressure monitoring. Approximately 30 min prior to anesthetic induction, ultrasound-guided regional blockade was administered, and the sensory distribution of the block was reassessed 20 min later to confirm anesthetic efficacy. Induction of general anesthesia was achieved with intravenous administration of etomidate (0.2 mg/kg), midazolam (0.03 mg/kg), sufentanil (0.5 µg/kg), and vecuronium (0.1 mg/kg), ensuring optimal conditions for tracheal intubation. Anesthesia was subsequently maintained using a balanced intravenous–inhalational technique comprising continuous sevoflurane inhalation at 1%, propofol infusion at 2–4 mg/kg/h, remifentanil at 0.1–0.3 µg/kg/min, and vecuronium at 0.03 mg/kg/h (this protocol is a standardized procedure of our hospital, formulated in accordance with the principle of balanced anesthesia to reduce the dosage of single drugs and adverse reactions. It may not be a universally adopted regimen worldwide and could limit the external validity (generalizability) of our results). Ventilatory settings were titrated to sustain end-tidal CO₂ between 35 and 45 mmHg, while the depth of anesthesia was guided by maintaining the bispectral index (BIS) within 40–60. Hemodynamic stability was strictly preserved, with heart rate and blood pressure controlled within ± 10 beats per minute and ± 20% of baseline values, respectively. At the conclusion of surgery, patients remained intubated and were transferred to the post-anesthesia care unit (PACU) for further monitoring and recovery management. Muscle relaxant antagonists (sugammadex sodium 2 mg/kg), flumazenil 0.2 mg (Flumazenil should be administered only when the patient’s consciousness has not been restored (e.g., unresponsiveness to calls) prior to extubation, with an additional 0.1 mg if necessary, aiming to accelerate consciousness recovery and reduce awakening delay) were used. Tubes were extubated when extubation criteria were met, and patients were transferred to the ward when the Aldrete recovery score was ≥ 9. All patients received 50 mg flurbiprofen axetil intravenously at the end of surgical suturing and postoperative intravenous PCA: 2 µg/kg sufentanil citrate + 6 mg tropisetron hydrochloride + normal saline (total 120 mL). PCA parameters: background infusion 2 mL/h, bolus dose 0.5 mL, initial dose 0 mL (The PCA initial dose is set to 0 mL, meaning no baseline auto-infusion is initiated after patient awakening. A 0.5 mL bolus dose must be triggered by manual button press, with a lockout time of 15 min), lockout time 15 min. If the NRS score was ≥ 4 points, the PCA pump was pressed; if pain was uncontrolled, intravenous injection of 5 mg dezocine as rescue analgesia until the NRS score < 3 points (Maximum dose within 24 h ≤ 20 mg (to avoid the risk of respiratory depression)).

### Ultrasound-guided RSSB and TAPB

All blocks were performed by an experienced anesthesiologist. Instruments: 22G 200 mm ultrasound block needle (Stimuplex, B. Braun Aesculap Japan Co., Ltd.), ultrasound system (Model: S9, Shenzhen Mindray Biomedical Electronics Co., Ltd.), low-frequency convex array probe, and high-frequency linear array probe.

**Fig. 1:**
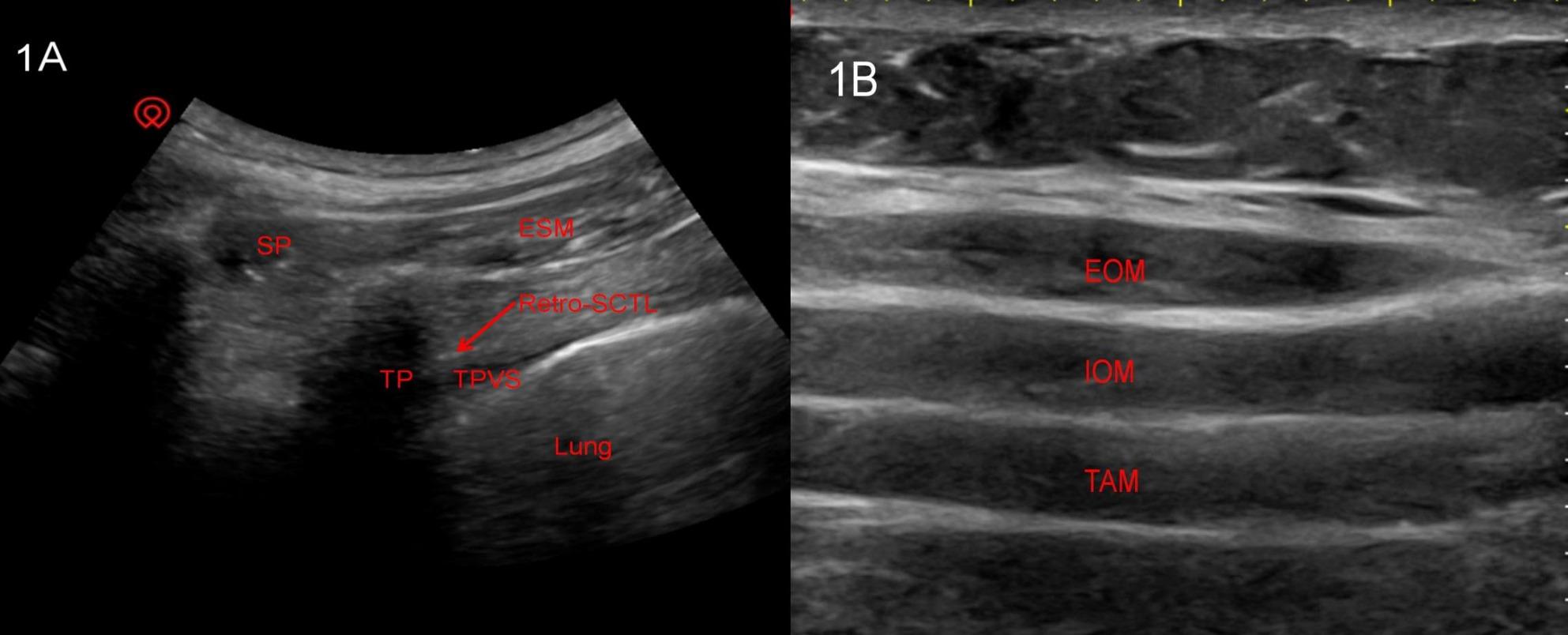
A comparison of two sets of ultrasound images. 1**A** Ultrasound findings of the posterior interspinal block site in the supraspinous ligament. *Abbreviations*: *SCTL* superior costotransverse ligament, *ESM* erector spinae muscle, *TP* transverse process, *TPVS* Thoracic Paravertebral space, *SP* Spinous Process. 1**B** Transversus abdominal muscle plane ultrasound findings. *Abbreviations*: *TAM* Transversus Abdominal Muscle, *EOM* External Oblique Muscle, *IOM* Internal Oblique Muscle

Retro-Superior Costotransverse Ligament Space Block: thirty minutes before anesthesia induction, the patient was placed in the right lateral position, and a portable color Doppler ultrasound system equipped with a low-frequency convex array probe was utilized for transverse imaging. First, the thoracic vertebral segment was identified by counting ribs and transverse processes, with the T7–8 segment marked on the body surface. The probe was then moved caudally to the transverse process level to visualize the thoracic paravertebral space (TPVS), followed by probe adjustment to clearly identify the inferior articular process (IAP) and further optimization of the viewing angle to distinctly display the superior costotransverse ligament (SCTL), IAP, and TPVS. Under real-time ultrasound guidance, a 20G 200 mm Stimuplex needle was inserted in-plane from lateral to medial, with the needle tip positioned in the hypoechoic retro-SCTL space (lateral to the IAP, Fig. 1A). After aspiration to confirm the absence of blood or air, 1 mL of normal saline was injected to observe space separation, and subsequently 20 mL of 0.375% ropivacaine hydrochloride (AstraZeneca, Sweden) was administered slowly. The aforementioned procedure was repeated on the contralateral side, with continuous monitoring for signs of local anesthetic systemic toxicity throughout the entire process.

Subcostal Transversus Abdominis Plane Block : Thirty minutes prior to anesthesia induction, the patient was placed in the supine position, and a portable color Doppler ultrasound system with a high-frequency linear array probe was employed for imaging. The probe was positioned vertically on the abdominal wall to scan the subcostal region along the anterior axillary line, targeting the anatomical plane between the external oblique muscle and transversus abdominis muscle (Fig. 1B). Under real-time ultrasound guidance, a 20G 200 mm Stimuplex needle was inserted in-plane from medial to lateral, with careful advancement to the predetermined target area. After aspiration to confirm the absence of blood or air, 1 mL of normal saline was injected to verify the separation between the posterior sheath of the rectus abdominis and the transversus abdominis muscle, followed by the slow administration of 20 mL of 0.375% ropivacaine hydrochloride. The entire procedure was replicated on the contralateral side to ensure bilateral analgesic coverage, with continuous monitoring for any signs of adverse reactions throughout the process.

### Laparoscopic gastrectomy

All procedures were conducted by the same experienced gastrointestinal surgical team, with patients positioned in the supine “split-leg” configuration to facilitate optimal abdominal access. Laparoscopic entry was achieved through five trocar ports strategically positioned according to the anatomical regions of the stomach. Pneumoperitoneum was established and maintained at an intra-abdominal pressure of 13.5–15 mmHg to ensure adequate visualization and operative space. Upon completion of the resection, the surgical specimen was retrieved via a 4–6 cm lower midline minilaparotomy. Following specimen extraction, two drainage catheters were routinely inserted through the subcostal trocar sites to prevent postoperative fluid accumulation and monitor intra-abdominal complications.

### Data collection and outcomes

Baseline data: Age, sex, BMI, comorbidities, Charlson Comorbidity Index (CCI), Intraoperative data: ASA classification, block duration, surgical type, surgical duration, drug/fluid/blood use, intraoperative blood loss, urine output.

Primary outcome: 24-hour postoperative resting NRS-AUC (assessed by blinded ward nurses at 1, 3, 6, 9, 12, and 24 h after surgery);

Secondary outcomes: Skin spread of the blocked nerve distribution area; Resting and movement NRS scores at 1, 3, 6, 9, 12, 24, 36 and 48 h after surgery (Movement was defined as three times of deep breath and cough once) [13]; 48-hour PCA use sufentanil dosage, number of patients requesting PCA bolus, time to first PCA bolus request, number of effective presses; Number of patients requesting rescue analgesia, time to first rescue analgesia, dezocine consumption; Overall Benefit of Analgesia Scale (OBAS) score; 1st postoperative day QoR-15 score; Postoperative recovery data (duration of first flatus, duration of first ambulation, duration of first oral liquid intake, duration of gastric tube removal); Incidence of postoperative nausea and vomiting (PONV); Length of hospital stay (LOS), incidence of non-surgical complications (pruritus, dizziness, respiratory depression, pneumothorax, hematoma, local anesthetic toxicity).

### Sample size and statistical analysis

Sample size estimation was conducted using PASS software version 15.0.5 (PASS, LLC, Kaysville, UT, USA). Preliminary data from a pilot cohort of 20 patients (10 per group) indicated that the 24-hour NRS area under the curve (AUC) was 54.3 ± 19.3 in the RSSB group and 84.2 ± 38.5 in the TAPB group. Based on these values, a two-tailed α of 0.05 and β of 0.10 (power = 90%) determined that 24 participants per group were required. Allowing for an anticipated dropout rate of 20%, a total of 60 patients (30 per group) were ultimately enrolled.

All statistical analyzes were performed using R software version 4.4.2 (R Foundation for Statistical Computing, Vienna, Austria). The area under the curve (AUC) of NRS scores was calculated using the trapezoidal rule with GraphPad Prism V.7 (GraphPad Software, San Diego, CA, USA) by multiplying time intervals by NRS scores. Normality of continuous variables was assessed using the Shapiro–Wilk test. 

Data conforming to a normal distribution were expressed as mean ± standard deviation (SD) and compared using independent-samples t-test, whereas non-normally distributed data were presented as median with interquartile range (IQR) and analyzed using the Mann–Whitney U test. Categorical variables were summarized as frequencies and percentages, and comparisons were conducted using the χ² test or Fisher’s exact test when expected cell counts were < 5. The P-value of < 0.05 was considered statistically significant. Hierarchical Bonferroni-Holm correction was used to control Type I error from multiple testing, with resting and movement NRS scores treated as independent test families and a significance level of α = 0.05.

## Results

**Fig. 2:**
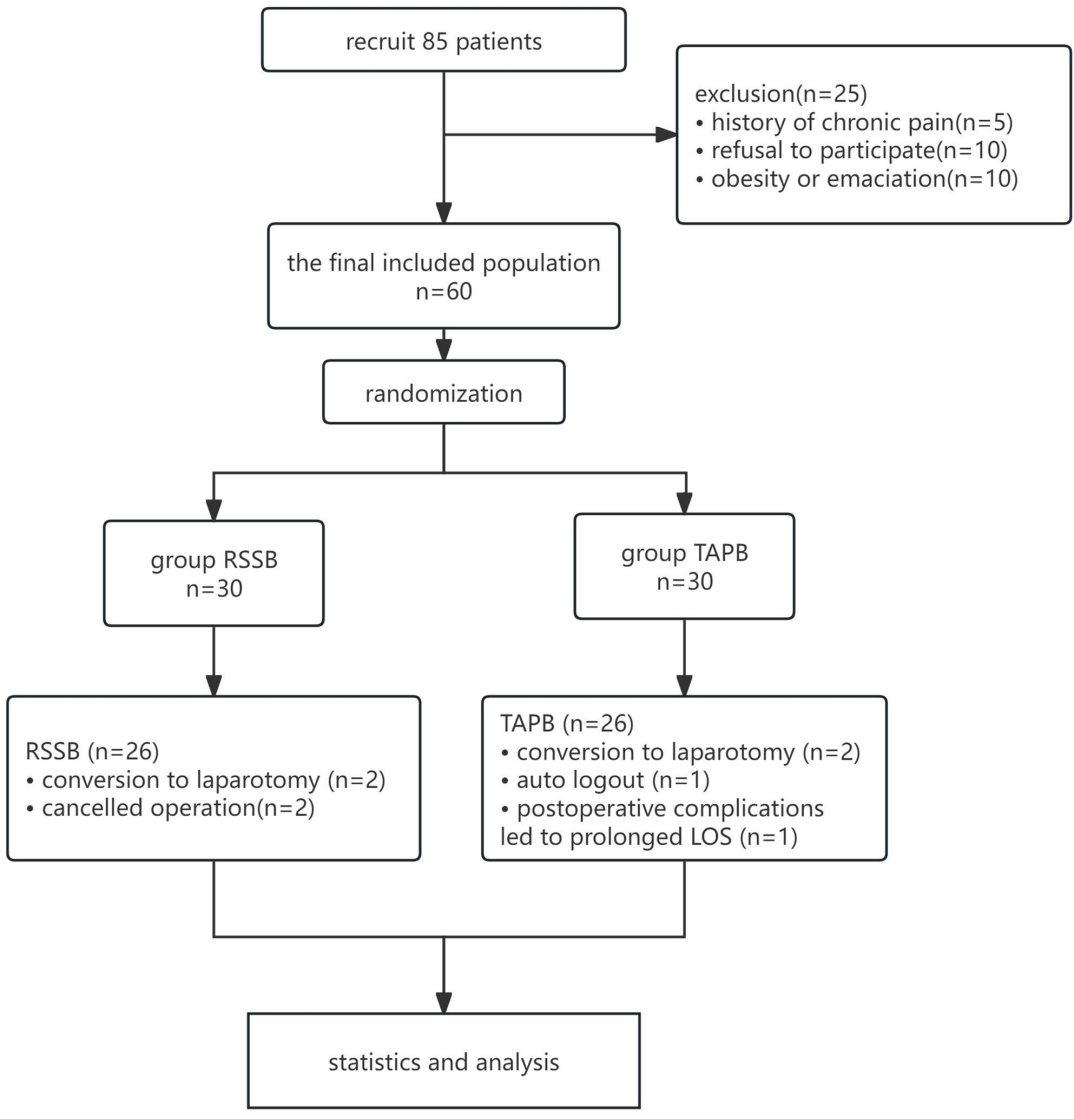
Patient flow chart. *Abbreviations*: *RSSB* Retro-Superior Costotransverse Ligament Space Block, *TAPB* Transversus Abdominis Plane Block, *LOS* Length of hospital Stay

Of 85 patients meeting inclusion criteria, 25 were excluded, and 60 were randomly assigned to the two groups. Eight patients withdrew after grouping, and 52 were finally included (Fig. 2). No patients were lost to follow-up. Demographic, anesthesia, and surgical data were comparable between the two groups (Table 1).

**Table 1:** Comparison of baseline characteristics, surgical and anesthetic data between the two groups

	Group TAPB (*n* = 26)	Group RSSB (*n* = 26)	*P*	95%CI	
				Min	Max
Baseline Characteristics					
Sex, male	17(65.4%)	17(65.4%)	> 0.999	-0.259	0.259
Age, yr	59.9 ± 11.9	55.8 ± 10.9	0.205	-2.300	10.450
BMI, kg/m^2^	23.4 ± 2.4	22.5 ± 2.3	0.179	-0.410	2.210
< 23.9	18(69.2%)	21(80.8%)			
24–27.9	8(30.8%)	5(19.2%)			
Comorbidity, n (%)					
Hypertension	10(38.5%)	3(11.5%)	0.025	1.034	10.741
Coronary heart disease	2(7.7%)	1(3.9%)	0.552	-0.088	0.165
Pulmonary diseases	1(3.9%)	0(0.0%)	> 0.999	-0.035	0.112
Diabetes	3(11.54%)	1(3.9%)	0.610	-0.066	0.220
Thyroid disease	2(7.7%)	1(3.9%)	0.552	-0.088	0.165
Hepatic diseases	1(3.9%)	2(7.7%)	0.552	-0.165	0.088
Renal diseases	1(3.9%)	0(0.0%)	> 0.999	-0.035	0.112
Anemia	10(38.5%)	9(34.6%)	0.773	-0.023	0.300
Cerebral vascular disease	3(11.5%)	0(0.0%)	0.234	-0.007	0.238
CCI	2(1,3)	1(0,2)	0.125	0.000	2.000
Hyponatremia, n(%)	2(7.7%)	1(3.9%)	0.552	-0.088	0.165
Hypoalbuminemia, n(%)	5(19.2%)	4(15.4%)	0.734	-0.120	0.197
Surgery and Anesthesia Characteristics					
Surgery Type, n (%)			0.795		
Total gastrectomy	4(15.4%)	4(15.4%)			
Distal gastrectomy	1(3.9%)	1(3.9%)			
Proximal gastrectomy	0(0.0%)	1(3.9%)			
Subtotal gastrectomy	21(80.8%)	20(76.9%)			
Surgery duration, min	212.5 ± 46.9	216.6 ± 52.5	0.765	-31.890	23.580
ASA Physical Status, n (%)			0.610		
II	23(88.5%)	25(96.1%)			
III	3(11.5%)	1(3.9%)			
Block duration, min	5(4,6)	4(2,6)	0.255	0.000	1.000
Intraoperative Medication					
Propofol, mg	540.4 ± 108.3	526.0 ± 148.4	0.691	-58.11	86.96
Sufentanil, µg	30.0(25.0,35,0)	30.0(24.5,35.5)	0.277	0.000	10.000
Remifentanil, µg	3.0 ± 0.8	3.1 ± 0.9	0.509	-0.650	0.250
Use of Vasopressors, n (%)					
Phenylephrine	21(80.8%)	23(85.2%)	0.701	-0.272	0.118
Ephedrine	12(46.2%)	15(57.7%)	0.579	-0.385	0.154
Atropine	2 (7.7%)	2(7.7%)	> 0.999	-0.145	0.145
Total infusion (mL)	2778.8 ± 509.7	2726.9 ± 397.1	0.684	-202.97	306.82
Use of Fluid					
Crystalloid, mL	2178.8 ± 447.0	2082.7 ± 381.3	0.408	-135.43	327.74
Colloid, mL	500.0(362.5,637.5)	500.00(350.0,650.0)	0.780	-200.0	100.0
Plasma transfusion, n (%)	1(3.9%)	3(11.5%)	0.603	-0.220	0.066
Red blood cell transfusion, n (%)	6(23.1%)	5(19.2%)	0.734	-0.183	0.260
Intraoperative blood loss (mL)	99.2 ± 74.9	64.0 ± 44.9	0.072	2.470	67.93
Intraoperative urine output (mL)	366.2 ± 354.0	369.6 ± 302.8	0.890	-179.13	172.33

Data are presented as mean ± standard deviation, median (IQR), or percentage (%)

*Abbreviations*: *BMI* Body Mass Index, *ASA* American Society of Anesthesiologists, *CCI* Charlson Comorbidity Index, *CI* Confidence Interval

**Fig. 3:**
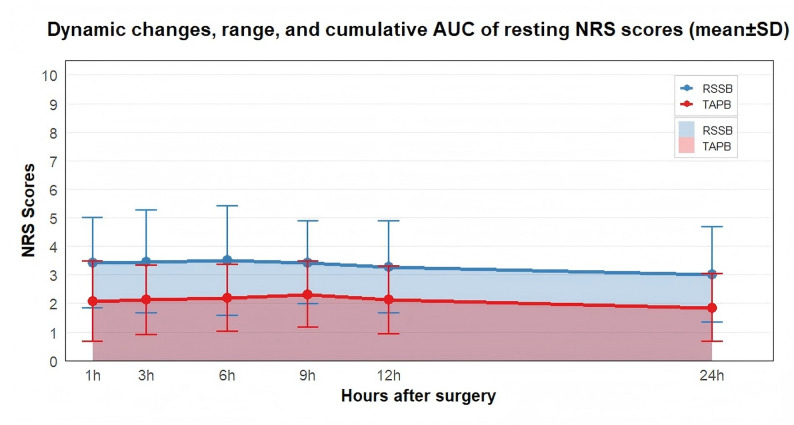
Dynamic changes, range, and cumulative AUC of resting NRS scores within 24 h after surgery. The lines represent the mean resting NRS scores of the RSSB and TAPB groups at each time point after surgery, with error bars indicating mean±standard deviation. The filled areas under the lines denote the cumulative area under the curve (AUC) of resting NRS scores within 24 h. The 24-hour resting NRS-AUC values were 48.3 ± 24.0 in the RSSB group and 75.4 ± 35.7 in the TAPB group. *Abbreviations*: *NRS *Numeric Rating Scale, *AUC* Area Under the Curve

The results showed that the 24-hour postoperative resting NRS-AUC was significantly lower in the RSSB group than in the TAPB group (mean (SD): 48.3 (24.0) vs. 75.4 (35.7); mean difference, 27.1; 95%CI: 10.69 to 44.42; *P* = 0.002; Fig. 3).

**Fig. 4:**
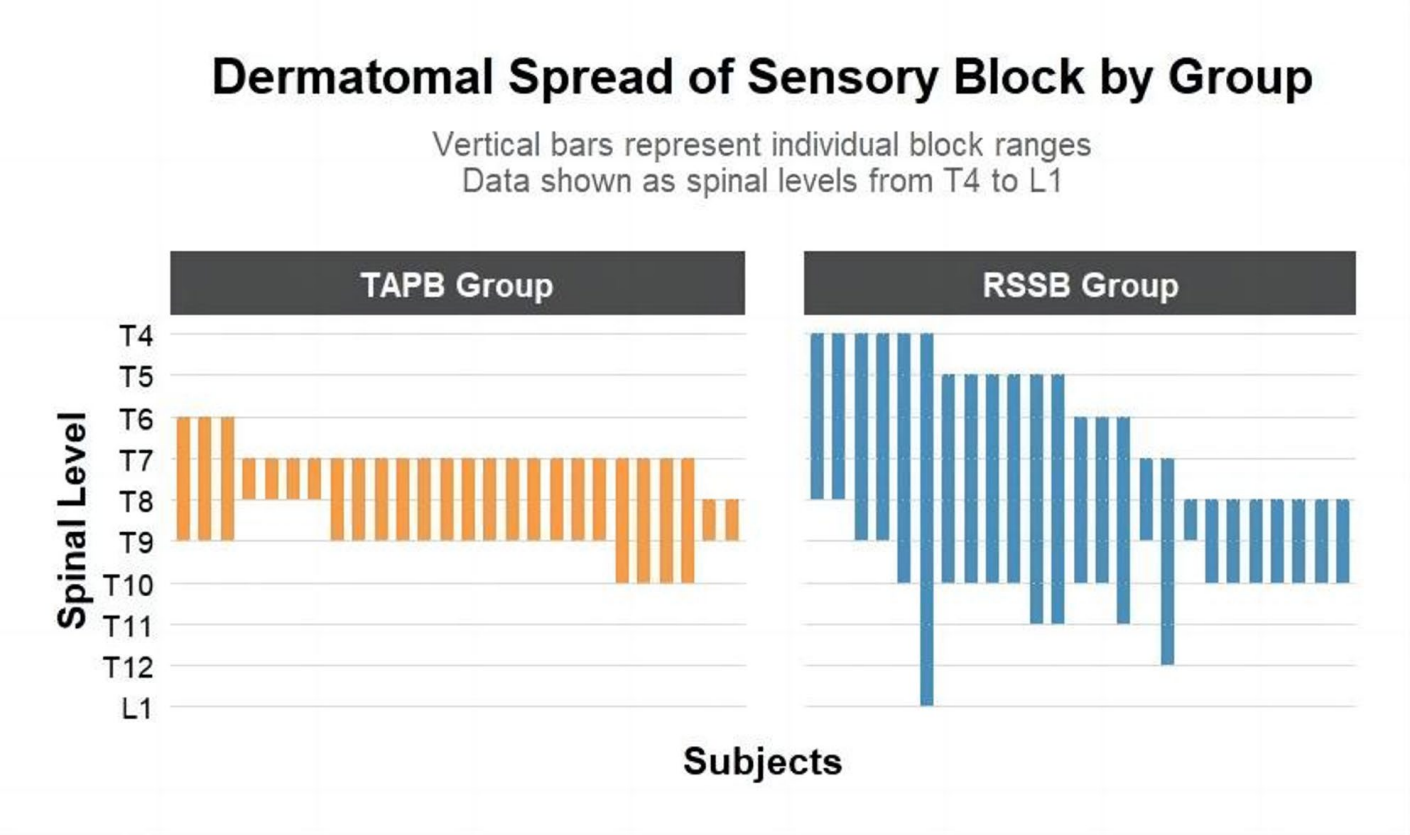
Stratified analysis of superficial skin block diffusion by nerve group. Cold sensation testing was conducted 20 min after block completion to evaluate diffusion patterns across dermatomes, performed along the midclavicular line. The bar chart illustrates the T4-L1 dermatome block area, representing the blocked dermatome range from the lowest to the highest level. Each bar indicates the block diffusion extent in individual subjects. The TAPB group (Transversus Abdominal Plane Block) showed 6 cases of single-segment cold sensation loss, while the RSSB group (Retro-Superior Costotransverse Ligament Space Block) had 1 case. *Abbreviations*: *TAPB* Transversus Abdominal Plane Block, *RSSB* Retro-Superior Costotransverse Ligament Space Block

The skin segmental spread range in the RSSB group was wider than that in the TAPB group. In the mid-clavicular region, the dermatomal count (from lowest to highest) was 5.2 ± 2.1 in the RSSB group and 3.0 ± 0.7 in the TAPB group (*P* < 0.001; Fig. 4).

**Fig. 5:**
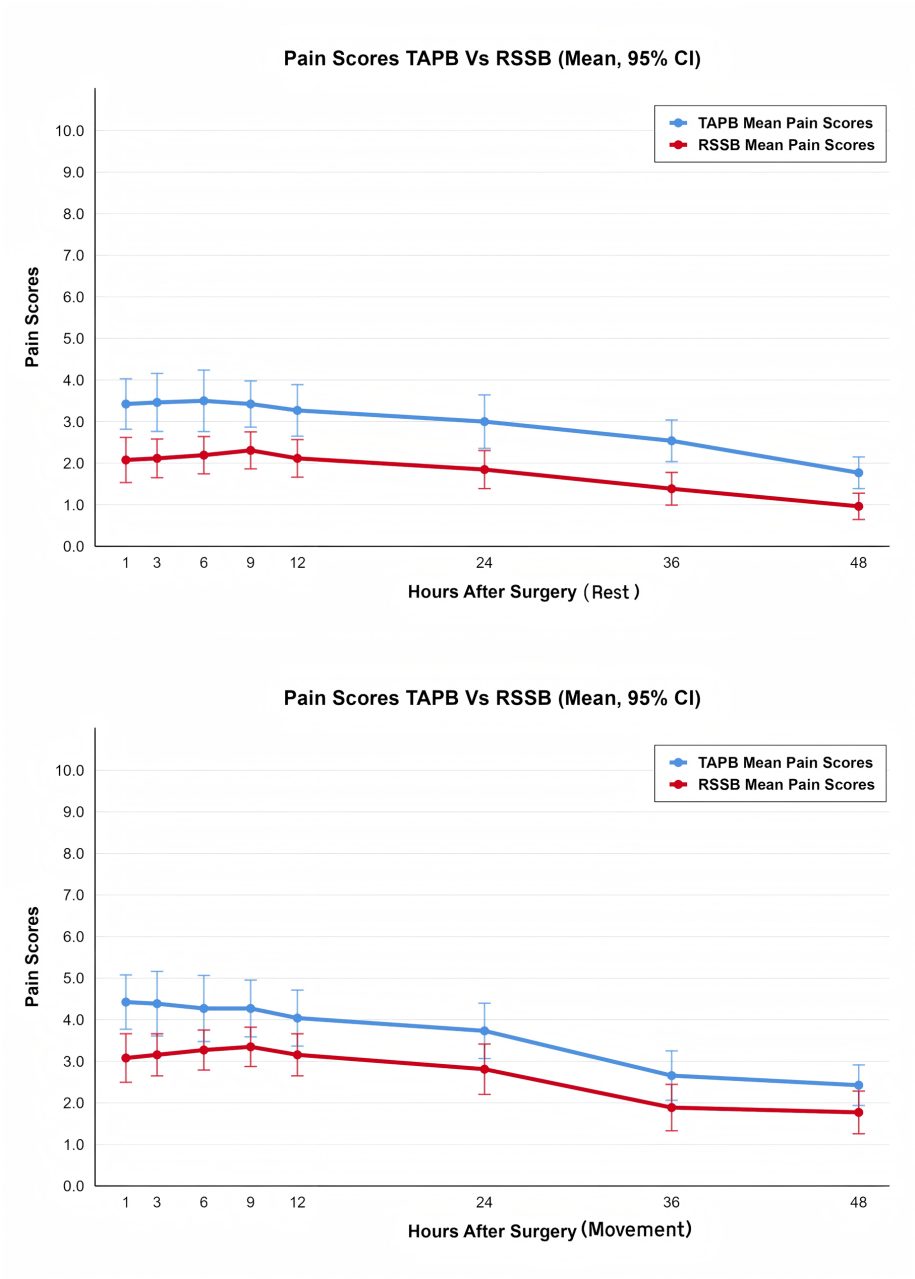
Resting and movement NRS scores at 1, 3, 6, 9, 12, 24,36 and 48 h after surgery. A: Resting pain scores assessed by numeric rating scale (NRS, 0–10) at 1, 3, 6, 9, 12, 24, 36, and 48 h after laparoscopic gastrectomy; B: Movement pain scores (defined as pain during three deep breaths plus one cough) assessed at the same time points. *Abbreviations*: *RSSB* Retro-Superior Costotransverse Ligament Space Block, *TAPB* Transversus Abdominis Plane Block, *NRS*  Numeric Rating Scale, *CI* Confidence Interval

The RSSB group had lower resting NRS scores at 1, 3, 6, 9, 12, 24, 36 and 48 h after surgery ( *P* < 0.05). Movement NRS scores were lower in the RSSB group at 1, 3, 6, and 9 h after surgery ( *P* < 0.05), but no significant differences were observed at 12, 24, 36, 48 h (Fig. 5). After correction, significant differences in resting NRS scores between the two groups were observed at all time points (all *P* < 0.05), while a significant difference in movement NRS scores was only noted at 1 h postoperatively (*P* = 0.003), with no statistical differences at other time points.

Regarding PCA, the time to first PCA bolus request was markedly prolonged in the RSSB group compared with the TAPB group (480.63 ± 385.80 vs. 249.06 ± 256.39 min, *P* = 0.034). No significant differences were detected between the groups in terms of total sufentanil consumption or the number of effective PCA attempts within 48 h. There were no statistically significant differences in either the cumulative dosage of dezocine or the frequency of additional analgesic interventions (Table 2).

**Table 2:** Secondary outcomes of postoperative analgesic use within 48 h

	Group TAPB (*n* = 26)	Group RSSB (*n* = 26)	*P*	95%CI	
				Min	Max
Analgesic medicine use (Postoperative Within 48 h)					
Sufentanil, µg	124.5(104.5,147.5)	117.5(107,130)	0.317	-8.000	20.000
Number of patients requesting PCA bolus, n(%)	16(61.5%)	19(73.1%)	0.375	-0.369	0.137
Time to first PCA bolus request, min*	249.1 ± 256.4	480.6 ± 385.8	0.034	-406.240	-56.760
Number of PCA bolus requests	4.7 ± 5.5	3.2 ± 3.3	0.231	-1.008	4.085
Number of patients requesting rescue analgesia, n (%)	13(50.0%)	11(42.3%)	0.578	-0.195	0.349
Time to first rescue analgesia, min	609.1 ± 667.6	544.3 ± 548.8	0.685	-260.720	389.320
Rescue Analgesic medicine consumption (dezocine, mg)	15.76 ± 9.54	16.81 ± 8.44	0.780	-5.870	3.770
OBAS	6.4 ± 1.9	5.9 ± 1.2	0.564	-0.340	1.340

Data are presented as mean ± standard deviation, median (IQR), or percentage

*Abbreviations*: *PCA* Patient-Controlled Analgesia, *QoR-15* Quality of Recovery-15 Scale, *OBAS* Overall Benefit of Analgesia Scale, *CI* Confidence Interval, *Min* lower bound of 95% confidence interval (CI), *Max* upper bound of 95% CI

In terms of overall recovery quality, the RSSB group demonstrated superior early postoperative recovery, evidenced by a higher QoR-15 score at 24 hours postoperatively (122.38 ± 7.08 vs. 114.96 ± 16.40, P = 0.039). Postoperative recovery parameters and safety profiles were comparable between the groups. No significant differences were observed in gastrointestinal recovery markers, including time to first flatus, ambulation, oral intake, or gastric tube removal. The incidence of PONV, length of hospital stay (LOS), and other non-surgical complications remained similar in both cohorts. Both groups received 40 mL of 0.375% ropivacaine (20 mL for each side), corresponding to a total dose of 150 mg. Based on the average body weight of the patients, this dose was far below the maximum recommended single dose of 3 mg/kg for ropivacaine. No local anesthetic systemic toxicity (LAST) reactions (e.g., perioral numbness, cardiac arrhythmias) were observed in either group. Only one case of Pruritus occurred in the RSSB group, which resolved spontaneously within 15 minutes (Table 3).

**Table 3:** Secondary outcomes of recovery quality, adverse events, and other outcomes

	Group TAPB (*n* = 26)	Group RSSB (*n* = 26)	*P*	95%CI	
				Min	Max
QoR-15 score of 24 h, points*	115.0 ± 16.4	122.4 ± 7.1	0.039	0.720	14.080
Duration of getting out of bed, d	1.9 ± 0.7	1.7 ± 0.7	0.184	-0.180	0.580
Duration of first flatus, d	2.8 ± 1.1	3.0 ± 1.1	0.352	-0.800	0.400
Duration of gastric tube removal, d	6.0 ± 6.3	5.4 ± 2.3	0.763	-1.900	3.100
Duration of first feed, d	6(4.0,6.8)	6(4.0,7.0)	0.723	-1.000	1.000
Length of hospital stay, d	14.5 ± 5.6	12.8 ± 2.8	0.295	-0.670	4.070
Postoperative adverse events, n (%)					
Postoperative nausea and vomiting	8(30.8%)	7(26.9%)	0.760	-0.208	0.285
Pruritus	0	1(3.9%)	> 0.999	-0.035	0.112
Dizzy	0	0			
Respiratory depression	0	0			
Pneumothorax	0	0			
Hematoma	0	0			
Local anesthetic toxicity	0	0			

Data are presented as mean ± standard deviation, median (IQR), or percentage

*Abbreviations*: *PONV* Postoperative Nausea and Vomiting, *LOS* Length of Stay, *CI* Confidence Interval, *Min* lower bound of 95% confidence interval (CI), *Max* upper bound of 95% CI

*P < 0.05, the difference is significant

## Discussion 

This is the first randomized controlled trial systematically comparing ultrasound-guided RSSB with subcostal TAPB in patients undergoing laparoscopic gastrectomy. Our results demonstrated that the RSSB group had a lower 24-hour postoperative resting NRS AUC, a longer time to the first PCA bolus request, and a higher QoR-15 score, indicating superior early postoperative analgesia and improved recovery quality.

Postoperative pain after laparoscopic gastrectomy includes somatic pain (related to abdominal wall incisions), visceral pain (sympathetically mediated), and inflammatory pain. With the promotion of the ERAS concept, nerve block combined with multimodal analgesia has been proven to enhance analgesic effects and promote early recovery. Thoracic epidural analgesia (TEA), although a traditional standard regimen for analgesia in abdominal surgery, carries risks of complications such as hematoma, hypotension, and urinary retention, which may delay patient recovery [11]. In recent years, several studies have proposed an ultrasound-guided technique—the "subcostal" TAPB—for providing reliable analgesia in supraumbilical abdominal surgeries [14]. However, subcostal TAPB can only relieve somatic pain and is insufficient in controlling visceral pain. Compared with paravertebral block or epidural block, its analgesic effect has limitations [8,11,14].

As a potential novel modified technique of TPVB, RSSB targets the retro-superior costotransverse ligament space, which was first proposed by Karmakar et al. This space behind the superior costotransverse ligament is extensively connected to the paravertebral space, intervertebral foramen, and erector spinae compartment, containing only loose connective tissue, as well as the posterior rami and proximal anterior rami of spinal nerves. In addition, the costotransverse space provides a potential vertical channel between the retro-SCTL space and the thoracic paravertebral (TPV) space of adjacent vertebrae [16]. Based on these anatomical studies, we hypothesized that RSSB would theoretically provide better analgesic effects than subcostal TAPB.

In this study, the RSSB group exhibited a significantly wider range of skin sensory block (P<0.001), and this advantage may be attributed to its anatomical characteristics: Cho et al. confirmed via micro-computed tomography (micro-CT) that the retro-superior costotransverse ligament (retro-SCTL) space communicates with the paravertebral space, intervertebral foramen, and erector spinae compartment, covering spinal nerve branches and the sympathetic trunk [15]; Karmakar et al. described the ultrasound visualization features of this space [16]. This anatomical connection supports the hypothesis that RSSB may act on both somatic and visceral nerve conduction simultaneously. However, due to the lack of a specific visceral pain assessment tool in this study, the independent analgesic effect on visceral pain cannot be clearly confirmed. Meanwhile, individual variations in the block range of RSSB (e.g., one patient with only single-segment block) may be related to the patency of the costotransverse space [15], the communication between the retro-SCTL space and the paravertebral space [17], the density of the intertransverse tissue complex [18], the sensitivity of the dorsal root ganglion [19], or puncture parameters [17]. Optimizing ultrasound-guided techniques and injection operations may reduce such variations.

In addition to the anatomical basis, we also verified the clinical significance of differences in NRS scores and NRS-AUC using recognized benchmarks [20,21]: a reduction of ≥2 points in NRS scores or ≥24 points·hours in AUC is considered clinically beneficial. The 24-hour postoperative NRS-AUC in the RSSB group was 27.1 points·hours lower than that in the TAPB group, and the resting NRS at 1 hour postoperatively (2.0 vs 4.0) and the movement NRS at 3 hours postoperatively (4.0 vs 6.0) both decreased by ≥2 points, confirming that the analgesic advantage of RSSB is not only statistically significant but also clinically applicable. This is consistent with the case report by Lee et al. [9] Meanwhile, combined with the conclusion reported by Chen et al. [11] that intertransverse process block is superior to TAPB, these results suggest that for upper abdominal surgeries, block techniques acting on the thoracic paravertebral region may have more significant analgesic advantages than TAPB limited to the abdominal wall.

Regarding safety, no serious complications (e.g., pneumothorax, local anesthetic toxicity) occurred in either group, with only 1 case of mild pruritus in the RSSB group—consistent with previous reports confirming RSSB's safety [17]. Additionally, RSSB significantly prolonged the time to first PCA bolus request, suggesting sustained early analgesia and reduced initial opioid dependence, despite no differences in 48-hour sufentanil consumption or effective PCA presses.For postoperative recovery, the RSSB group achieved higher 24-hour QoR-15 scores, reflecting improved multidimensional recovery (physiological comfort, psychological state, mobility). However, objective metrics (first out-of-bed time, flatus time, gastric tube removal, hospitalization duration) showed no significant differences, likely due to two factors: laparoscopic gastrectomy recovery is influenced by multiple confounders (surgical technique, nutrition, comorbidities), and our small sample size may have failed to detect subtle variations; objective outcomes are heavily standardized by postoperative care protocols, masking RSSB's potential benefits. These findings align with recent evidence on paraspinal blocks (e.g., erector spinae plane block, intertransverse process block) promoting postoperative recovery [12,22,23].

This study has limitations: first, this study is a single-center study with a relatively small sample size (60 cases initially, and 52 cases finally included). Although the calculation using PASS 15.0.5 software indicated that each group required at least 24 cases to achieve a 90% statistical power, there may still be Type II errors. These errors lead to the failure of some objective recovery indicators (such as length of hospital stay) to show statistical differences. Additionally, subgroup analysis was not conducted based on surgical methods (total gastrectomy, distal gastrectomy, proximal gastrectomy, subtotal gastrectomy), making it impossible to clarify the differences in the efficacy of RSSB across different surgical procedures. Second, this study has limitations related to blinding. The RSSB (side-lying position) and TAPB (supine position) are different postures. Although bias was reduced by standardized dressings and preoperative informed consent, only the assessors could be blinded, not the patients, which may affect the outcome measures. Meanwhile, all nerve blocks were performed by a single experienced anesthesiologist to control inter-operator variability and ensure procedural standardization. While this design enhanced internal validity by minimizing operator bias, it significantly restricted the generalizability of our results. Third, the follow-up of this study only lasted until the patients were discharged from the hospital. There is a lack of long-term follow-up data at 1 month, 3 months, etc. after surgery, which prevents the assessment of the impact of RSSB on patients' long-term quality of life (such as the incidence of chronic pain). 

Future research should focus on validating and expanding the clinical value of retro-superior costotransverse ligament space block (RSSB) through multiple key directions: conducting multicenter large-sample trials to confirm its efficacy across different laparoscopic gastrectomy subtypes (e.g., total vs. distal gastrectomy), further investigating optimal technical parameters such as comparing unilateral local anesthetic dosages (15 mL vs. 20 mL) and evaluating different thoracic injection segments (e.g., T6–7 vs. T7–8), performing comparative studies with established regional analgesic techniques (e.g., thoracic paravertebral block, epidural analgesia) to assess relative safety and efficacy, conducting long-term follow-up (3–6 months) to explore its potential role in preventing chronic postoperative pain, and involving multiple operators with varying skill levels to verify the consistency of RSSB's efficacy in real-world clinical scenarios and enhance the generalizability of research findings.

## Conclusions

Ultrasound-guided RSSB provides better postoperative analgesia than subcostal TAPB and improves early recovery quality for patients undergoing laparoscopic gastrectomy for gastric cancer, with favorable safety profiles. It is a promising option for perioperative analgesia in this patient population.

## Supplementary Information

Supplementary Material 1

## Data Availability

Due to concealment involving participants, de-identified datasets will be provided to qualified researchers upon reasonable request to the corresponding author.

## Abbreviations

RSSB: Retro-superior costotransverse ligament space block
TAPB: Transversus abdominis plane block
TPVB: Thoracic paravertebral block
ESM: Erector Spinae Muscle
TP: Transversus Process
TPVS: Thoracic Paravertebral space
IAP: Inferior Articular Process
SP: Spinous Process
TAM: Transverse Abdominal Muscle
EOM: External Oblique Muscle
IOM: Internal Oblique Muscle
NRS: Numeric rating scale
AUC: Area under the curve
PCA: Patient-controlled analgesia
ASA: American Society of Anesthesiologists
BMI: Body mass index
CI: Confidence Interval
SD: Standard deviation
IQR: Interquartile range
