# Supplementary material for: Ultrasound-guided retro-superior costotransverse ligament space block (RSSB) versus subcostal transversus abdominis plane block (TAPB) for postoperative analgesia in gastric cancer patients undergoing laparoscopic gastrectomy : a prospective randomized controlled trial
Source: BMC Anesthesiol. 2026 Feb 23;26:199. doi: 10.1186/s12871-026-03700-7 (PMC13037083; doi:10.1186/s12871-026-03700-7)
Supplement: Supplementary file 1 — Supplementary Material 1 [file 12871_2026_3700_MOESM1_ESM.docx]

Table 4. Heart rate and mean arterial pressure (MAP) at five time points during the procedure.

|  | HR, bpm |  |  |  | MAP, mmHg |  |  |  |
| --- | --- | --- | --- | --- | --- | --- | --- | --- |
| time point | TAPB group (n = 26) | RSSB group (n = 26) | P price | 95% CI | TAPB group (n = 26) | RSSB group (n = 26) | P price | 95% CI |
| base line | 73.12 ± 11.417 | 73.58 ± 12.407 | 0.890 | −7.103,6.180 | 95.86 ± 10.717 | 101.38 ± 11.522 | 0.079 | −11.726,0.671 |
| After induction | 59.92 ± 12.221 | 61.38 ± 12.541 | 0.672 | −8.359,5.436 | 81.05 ± 9.931 | 80.50 ± 10.155 | 0.843 | −5.042,6.149 |
| After the skin incision | 58.85 ± 11.415 | 56.85 ± 9.490 | 0.495 | −3.847,7.847 | 91.91 ± 13.116 | 85.82 ± 12.637 | 0.094 | −1.086,13.263 |
| After skin suturing | 60.88 ± 12.810 | 61.58 ± 11.649 | 0.839 | −7.513,6.128 | 83.33 ± 10.813 | 84.09 ± 10.809 | 0.801 | −6.781,5.264 |
| During intubation removal | 75.42 ± 12.558 | 75.77 ± 15.167 | 0.929 | −8.102,7.410 | 108.00 ± 13.127 | 105.86 ± 12.809 | 0.554 | −5.083,9.367 |

Data are presented as mean±SD.

MAP: Mean Arterial Pressure; HR: Heart Rate; CI: confidence interval.

Table 5. Multiple sensitivity analysis table

| Model (adjust variables) | between-group differences（B） | 95%CI | P-value | R² |
| --- | --- | --- | --- | --- |
| Model 1: Unadjusted | -27.1 | -44.4 to -10.7 | 0.002 | 0.178 |
| Model 2: +hypertension | -24.3 | -41.9to-6.7 | 0.008 | 0.202 |
| Model 3: +Hypertension +Age | -24.2 | -42.4 to-6.2 | 0.009 | 0.202 |

present the estimated treatment effects (β coefficients) and their 95% confidence intervals under different adjustment schemes

Note: A negative B-value indicates that the NRS-AUC in the RSSB group is lower than that in the TAPB group.
